# *LPS1*, Encoding Iron-Sulfur Subunit SDH2-1 of Succinate Dehydrogenase, Affects Leaf Senescence and Grain Yield in Rice

**DOI:** 10.3390/ijms22010157

**Published:** 2020-12-25

**Authors:** Chun Li, Chuan-Qiang Liu, Hong-Shan Zhang, Cong-Ping Chen, Xiao-Rong Yang, Long-Fei Chen, Qing-Song Liu, Jia Guo, Chang-Hui Sun, Ping-Rong Wang, Xiao-Jian Deng

**Affiliations:** 1 State Key Laboratory of Crop Gene Exploration and Utilization in Southwest China, Sichuan Agricultural University, Chengdu 611130, China; lichun99@stu.sicau.edu.cn (C.L.); 13855@sicau.edu.cn (C.-H.S.); 2 Rice Research Institute, Sichuan Agricultural University, Chengdu 611130, China; chuanqiangliu4@gmail.com (C.-Q.L.); hszhang123@gmail.com (H.-S.Z.); chen1564654378@gmail.com (C.-P.C.); yangxiaorong@stu.sicau.edu.cn (X.-R.Y.); ornmonetrcqvy11@gmail.com (L.-F.C.); 2019311050@stu.sicau.edu.cn (Q.-S.L.); guojia6@stu.sicau.edu.cn (J.G.)

**Keywords:** *Oryza sativa*, succinate dehydrogenase, iron-sulfur subunit, ROS, late premature senescence, panicle development, grain yield

## Abstract

The iron-sulfur subunit (SDH2) of succinate dehydrogenase plays a key role in electron transport in plant mitochondria. However, it is yet unknown whether *SDH2* genes are involved in leaf senescence and yield formation. In this study, we isolated a late premature senescence mutant, *lps1*, in rice (*Oryza sativa*). The mutant leaves exhibited brown spots at late tillering stage and wilted at the late grain-filling stage and mature stage. In its premature senescence leaves, photosynthetic pigment contents and net photosynthetic rate were reduced; chloroplasts and mitochondria were degraded. Meanwhile, *lps1* displayed small panicles, low seed-setting rate and dramatically reduced grain yield. Gene cloning and complementation analysis suggested that the causal gene for the mutant phenotype was *OsSDH2-1* (*LOC_Os08g02640*), in which single nucleotide mutation resulted in an amino acid substitution in the encoded protein. *OsSDH2-1* gene was expressed in all organs tested, with higher expression in leaves, root tips, ovary and anthers. OsSDH2-1 protein was targeted to mitochondria. Furthermore, reactive oxygen species (ROS), mainly H_2_O_2_, was excessively accumulated in leaves and young panicles of *lps1*, which could cause premature leaf senescence and affect panicle development and pollen function. Taken together, *OsSDH2-1* plays a crucial role in leaf senescence and yield formation in rice.

## 1. Introduction

Known as the powerhouses of the eukaryotic cell, mitochondria provide usable energy for the cell′s survival and functioning. Succinate dehydrogenase (SDH), also called mitochondrial complex II, is the only enzyme participating in tricarboxylic acid (TCA) cycle as well as the electron transport chain (ETC), generating ATP for cells [1,2]. SDH catalyzes the oxidation of succinate to fumarate in the TCA cycle, and the electrons generated in the oxidation reaction are transferred into the ETC, reducing the ubiquinone into ubiquinol [3,4,5].

SDH is made up of four classical subunits: SDHA-SDHD in *Escherichia coli* and animals [4,5], and SDH1-SDH4 in yeast and plants [6,7,8]. SDH1/SDHA is a flavoprotein including a substrate binding site and a flavin adenine dinucleotide (FAD) cofactor. SDH2/SDHB is an iron-sulfur (Fe-S) protein harboring three Fe-S clusters. SDH3/SDHC and SDH4/SDHD are two hydrophobic membrane proteins including the ubiquinone binding and reduction site [4,5]. SDH plays a critical role in mitochondrial metabolism. In animals and humans, mutations in SDH subunits result in various diseases, for instance, ageing in nematodes [9] and tumors in humans including paraganglioma-pheochromocytoma, gastrointestinal gtromal tumor, renal cell carcinoma and pituitary pdenoma [2,10].

In *Arabidopsis thaliana*, the flavoprotein subunit (SDH1) is encoded by two genes (*SDH1-1* and *SDH1-2*). *SDH1-1* RNA interference line is reported to display pollen abortion and a reduced seed set [11]. In addition, *disrupted stress response 1* (*dsr1*) mutant with a mutation at the succinate binding site of SDH1-1 shows reduced SDH activity, lowered mitochondrial ROS (reactive oxygen species) production and decreased expression levels of early stress responsive gene [12]. For iron-sulfur protein subunit (SDH2), three nuclear genes *AtSDH2-1*, *AtSDH2-2* and *AtSDH2-3* encode this subunit [11,13,14]. No abnormal phenotype is observed in *SDH2-1* or *SDH2-2* mutant with T-DNA insertion, indicating that *SDH2-1* and *SDH2-2* are redundancy genes [14,15]. The knockout of *SDH2-3* results in delayed seed germination, suggesting that the role of *SDH2-3* at an early step of seed germination [16]. In tomato (*Solanum lycopersicum*), SDH2 is encoded by two genes (*SlSDH2-1* and *SlSDH2-2*), and *SlSDH2-2* RNA interference line exhibits increased photosynthetic rate and enhanced growth [17]. However, it is yet unclear the impact of *SDH* defect on the growth and development of monocotyledonous plants.

In rice (*Oryza sativa*), SDH2 is encoded by two genes, *OsSDH2-1* (*LOC_Os08g02640*) and *OsSDH2-2* (*LOC_Os09g20440*), respectively [7,18]. Interestingly, the *LOC_Os08g02640* gene is a chimeric gene and encodes SDH2 and chimeric SDH2-RPS14 (ribosomal protein S14) by alternative splicing [19]. Compared to the functional RPS14, an additional fragment with 250 amino acids is located at the N-terminal region of SDH2-RPS14 protein, and position 1–225 amino acids are identical to the corresponding sequences of OsSDH2-1, providing the mitochondrial signal peptide for RPS14 [19]. A similar chimeric *SDH2*-*RPS14* gene also has been found in maize [20,21]. Nevertheless, no *sdh2* mutant has been identified in monocotyledonous plants.

In this study, we isolated a *late premature senescence* mutant, *lps1*, in rice. The mutant showed normal growth at seedling stage, but its leaves exhibited brown spots at the late tillering stage and wilted at the late grain-filling stage and mature stage. Furthermore, *lps1* displayed small panicles, low seed-setting rate and dramatically reduced grain yield. Through gene mapping, MutMap analysis and complementation assay, we identified that the *OsSDH2-1* (*LOC_Os08g02640*) gene was responsible for the mutant phenotype of *lps1*. Reactive oxygen species (ROS) were accumulated in leaves and young panicles of *lps1*. *OsSDH2-1* gene was ubiquitously expressed with high expression levels in leaves, root tips and floral organs, and its encoded protein was localized to the mitochondria. In addition, we examined expression difference of three senescence-associated genes and three ROS detoxification-associated genes between *lps1* and wild type. Our study suggested that *SDH2-1* plays a crucial role in leaf senescence and yield formation in rice.

## 2. Results

### 2.1. Late Premature Senescence and Dramatically Reduced Grain Yield in Lps1

**Figure 1:**
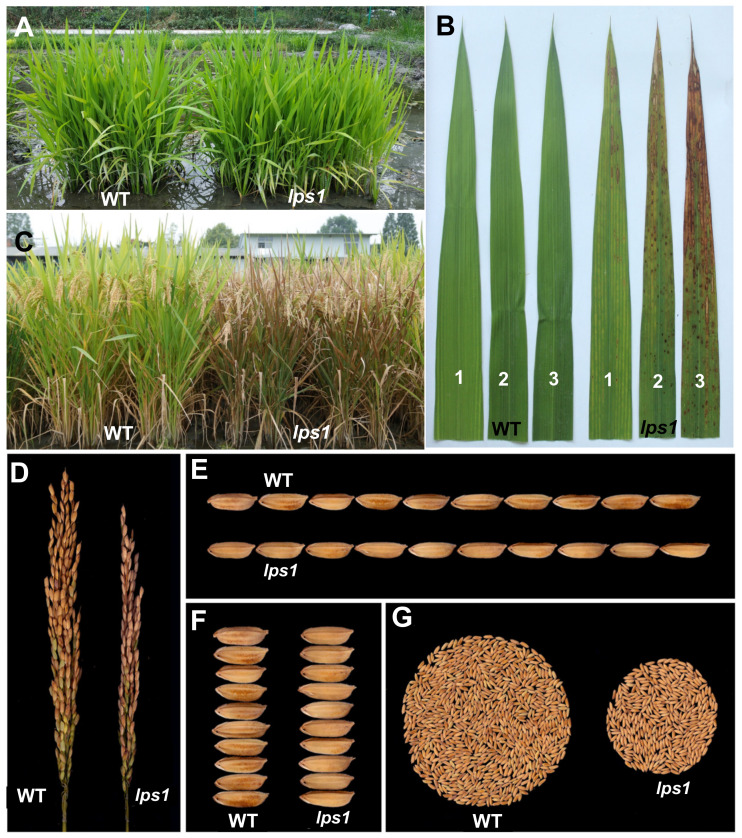
Phenotypic comparison between wild type (WT) and *lps1* mutant. (**A**) The field phenotype at seedling stage. (**B**) The top three leaves at heading stage. One, 2 and 3 indicate flag leaf, the 2nd leaf and the 3rd leaf from the top, respectively. (**C**) The field phenotype at mature stage. (**D**) Panicle morphology. (**E**) Grain length. (**F**) Grain width. (**G**) Grain yield per plant.

We isolated a late premature senescence mutant, *lps1*, from a mutant pool of main restorer line Lehui188 (188R, *indica*) via ethyl methanesulfonate (EMS) mutagenesis. At seedling stage, the mutant exhibited normal phenotype as its wild type (Figure 1A). At the late tillering stage, brown spots were firstly initiated from the tip of lower leaves of *lps1* and then gradually spread to whole leaves (Figure 1B,C). On the other hand, top-one leaves did not show any brown spots when they just full-expanded. Thereafter, brown spots appeared at the tip of top-one leaves (Figure 1B). At the late grain-filling stage and mature stage, almost all leaves of *lps1* wilted, while the upper leaves of wild type displayed normal yellow-green color (Figure 1C).

**Figure 2:**
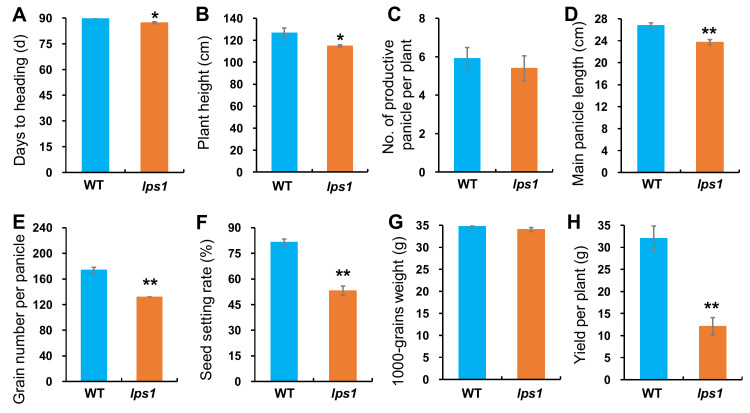
Comparison of major agronomic traits between WT and *lps1*. Error bars represent standard deviations of three independent biological replicates. (**A**) Days to heading. (**B**) Plant height. (**C**) Number of productive panicles per plant. (**D**) Main panicle length. (**E**) Grain number per panicle. (**F**) Seed setting rate. (**G**) 1000-grains weight. (**H**) Grain yield per plant. The statistically significant differences were performed by Student’s *t* test. * *p* < 0.05 and ** *p* < 0.01.

Investigation of agronomic traits demonstrated that there was no significant difference in number of productive panicles, 1000-grains weight, grain length and width between *lps1* and wild type (Figure 1E,F, Figure 2C,G and Supplementary Figure S1), while *lps1* were significantly reduced in days to heading (−3.0%), plant height (−9.3%), main panicle length (−11.5%), grain number per panicle (−24.1%) and seed-setting rate (−28.3%) compared with wild type (Figure 1C,D and Figure 2A,B,D–F). Taken together, reduced grain number per panicle and seed-setting rate resulted in dramatically reduced grain yield by 62.3% in *lps1* (Figure 1D,G and Figure 2H).

### 2.2. Decreased Photosynthetic Capacity in Lps1 

**Figure 3:**
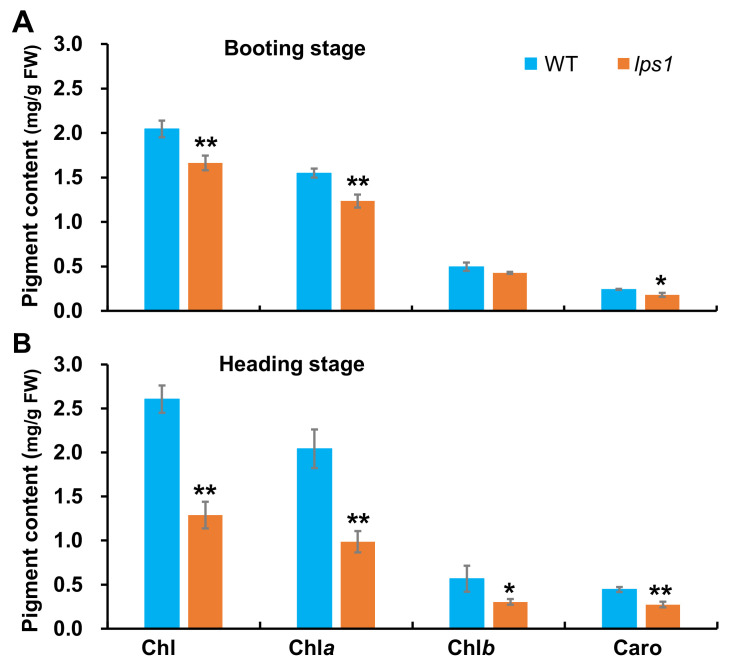
Pigment contents in leaves of WT and *lps1*. (**A**) The top leaf at booting stage. (**B**) The flag leaf at heading stage. Error bars represent standard deviations of three independent biological replicates. The statistically significant differences were performed by Student’s *t* test. * *p* < 0.05 and ** *p* < 0.01.

In order to investigate influence of *LPS1* mutation on photosynthetic pigments, we quantified pigment contents of the top-one leaves at booting stage and the flag leaves at heading stage. As shown in Figure 3, contents of pigments including total chlorophyll (Chl), Chl *a*, Chl *b* and carotenoids in *lps1* were all reduced by 18.7, 20.1, 13.7 and 25.0%, respectively, compared to those in wild type at booting stage. At heading stage, the pigment contents in *lps1* continued to reduce, and decreased by 50.6, 51.7, 46.4 and 38.7%, respectively, compared to those in wild type. The results suggested that the pigment contents of *lps1* was remarkably affected by the mutation of *LPS1*.

**Figure 4:**
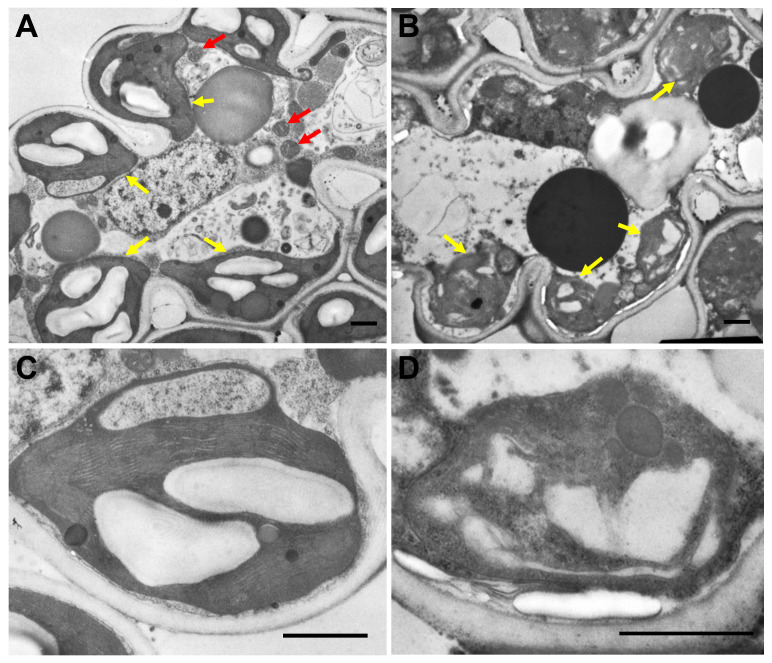
Ultrastructure of chloroplasts in mesophyll cells of flag leaves showing premature senescence phenotype at heading stage. (**A**,**B**) Mesophyll cell of the wild type and *lps1*, respectively. (**C**,**D**) Chloroplast structure of the wild type and *lps1*, respectively. Red arrows indicate mitochondria and yellow arrows indicate chloroplasts. Bar = 1 μm.

Subsequently, we observed mesophyll cells of the flag leaves showing premature senescence phenotype at heading stage using transmission electron microscope. In the wild type, chloroplasts showed regular shape, their envelope membranes had distinct boundaries and the thylakoid membrane structure and grana stacks could be obviously observed (Figure 4A,C). By contrast, in mesophyll cells of *lps1*, chloroplasts had no intact structure, their envelop membranes were destroyed, and the thylakoid membrane structure was completely lacked in only a few visible chloroplasts (Figure 4B,D). On the other hand, many mitochondria with normal structure were observed in the wild-type mesophyll cells (Figure 4A), while almost no mitochondria with normal size have been found in the mutant mesophyll cells (Figure 4B), implying that mitochondria in the mutant were highly deformed even if they might still be present. The data suggested that both chloroplasts and mitochondria were degraded in these leaves of *lps1*.

**Figure 5:**
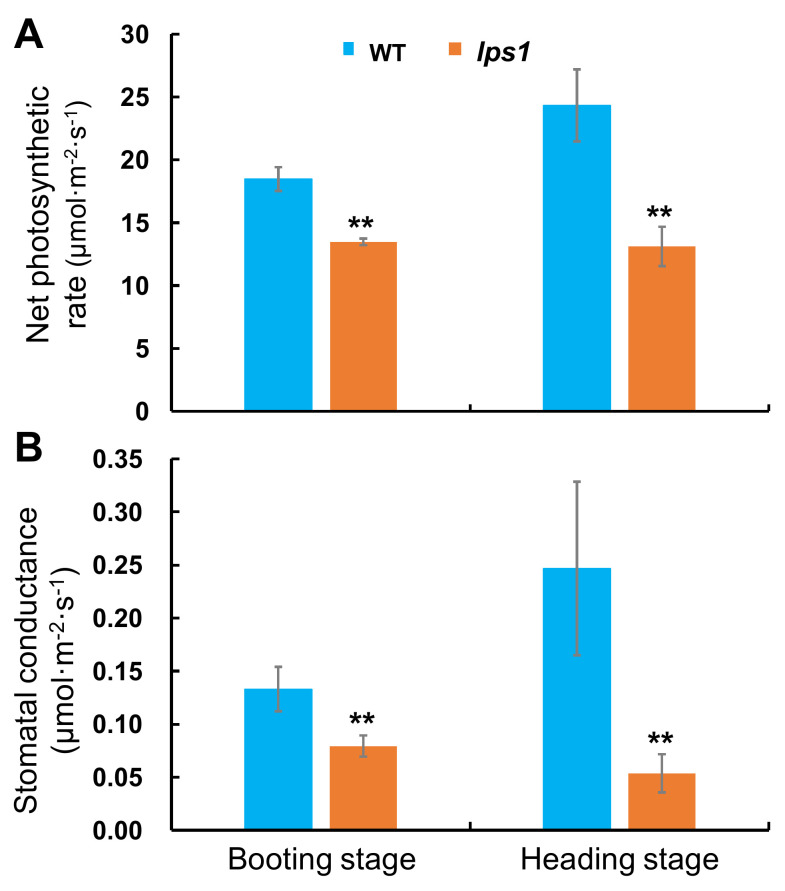
Photosynthetic capacity of top one leaf or flag leaf of WT and *lps1* at booting stage and heading stage. (**A**) Net photosynthetic rate (*P*_n_). (**B**) Stomatal conductance (*G*_s_). Error bars represent standard deviations of three independent biological replicates. The statistically significant differences were performed by Student’s *t* test. ** *p* < 0.01.

To further investigate whether photosynthetic capacity of *lps1* was affected, we measured photosynthetic parameters including net photosynthetic rate (*P*_n_) and stomatal conductance (*G*_s_) at booting and heading stages, respectively. As expected, net photosynthetic rate and stomatal conductance of *lps1* were dramatically reduced by 27.0 and 40.3%, respectively, at booting stage, and 46.1 and 78.3%, respectively, at heading stage compared to those of wild type (Figure 5). Our data suggested that the photosynthetic capacity of *lps1* was pronouncedly reduced due to the mutation of *LPS1*.

### 2.3. LPS1 Encodes Iron-Sulfur Subunit SDH2-1 of Succinate Dehydrogenase

For genetic analysis of the mutant, F_1_ and F_2_ populations were constructed by crossing *lps1* and wild type. All F_1_ plants exhibited normal phenotype as wild type. Additionally, the segregation ratio of F_2_ plants with wild-type phenotype to those with late premature senescence phenotype demonstrated a good fit to 3:1 (χ^2^ = 0.54 < χ^2^_0.05_ = 3.84). The results suggested that the late premature senescence phenotype of *lps1* was governed by a single recessive nuclear gene.

To perform preliminary mapping of *lps1* locus, 116 plants with mutant phenotype were selected from F_2_ population crossing between *lps1* mutant and *japonica* variety Nipponbare. The preliminary mapping result revealed that *lps1* gene was located on the short arm of chromosome 8 and linked with RM1019, at genetic distance of 6.6 cM. Then, three new InDel markers (L1, L2 and L3) were developed to carry out linkage analysis, and *lps1* locus was finally mapped to a 1460 kb region between InDel markers L1 and L3 (Supplementary Figure S2).

Subsequently, the MutMap method was used to identify the candidate gene with 30 plants showing mutant phenotype from (*lps1*×188R) F_2_ population. The results showed that only one sense mutation occurred in the 1460 kb region, which carried a single nucleotide C-to-T substitution at position 412 in *LOC_Os08g02640* genome sequence. *LOC_Os08g02640* encodes the iron-sulfur subunit of succinate dehydrogenase (SDH2) and the ribosomal protein S14 (SDH2-RPS14) by alternative splicing, respectively [19]. Then, we amplified and sequenced the two transcripts with wild-type cDNAs, confirming that *LOC_Os08g02640* gene does have these two alternative splice forms. cDNA sequencing analysis of *lps1* mutant revealed that a C-to-T substitution happened at codon 412 in the coding sequence of *LOC_Os08g02640*, resulting in an amino acid substitution from Leu-138 to Phe. Thereby, *LOC_Os08g02640* was selected as the candidate gene for the mutant phenotype of *lps1*.

*LOC_Os08g02640* gene is a full-length of 1793 bp genomic DNA and has two different alternative splice forms, corresponding to two different gene structure (Supplementary Figure S3). For *OsSDH2-1* alternative splice form, *LOC_Os08g02640* gene comprised two exons and one intron with 846 bp cDNA and encoded a protein of 281 amino acids with a 25 amino acid mitochondrial signal peptide at N-terminus (Supplementary Figure S3). For *SDH2-RPS14* alternative splice form, *LOC_Os08g02640* gene comprised two exons and one intron with 1053 bp cDNA and encoded a protein of 350 amino acids with a common mitochondrial signal peptide at N-terminus (Supplementary Figure S3). In addition, according to the results of Huang et al. [18] and blastP with amino acid sequence of OsSDH2-1 encoded by *LOC_Os08g02640* in Rice Annotation Project Database, there was also a homologous gene, *OsSDH2-2* (*LOC_Os09g20440*), in rice genome.

**Figure 6:**
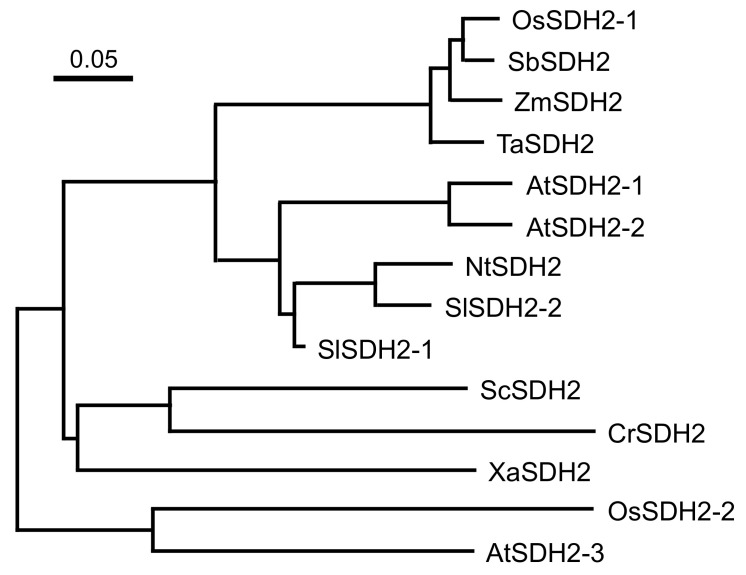
Phylogenetic analysis of OsSDH2-1 and its homologs. Accession numbers in Uniprot for the respective protein sequences are as follows: OsSDH2-1 (*Oryza sativa*, LOC_Os08g02640.2), OsSDH2-2 (*Oryza sativa*, LOC_Os09g20440.1) TaSDH2 (*Triticum aestivum*, A0A3B6RH41), ZmSDH2 (*Zea mays*, B4FJ16), SbSDH2 (*Sorghum bicolor*, A0A1Z5R8P3), AtSDH2-1 (*Arabidopsis thaliana*, Q8LBZ7), AtSDH2-2 (*Arabidopsis thaliana*, Q8LB02), AtSDH2-3 (*Arabidopsis thaliana*, Q9FJP9), SlSDH2-1 (*Solanum lycopersicum*, A0A3Q7FBI5), SlSDH2-2 (*Solanum lycopersicum*, D2KQI9), NtSDH (*Nicotiana tabacum*, A0A1S4AWC1), XaSDH2 (*Xanthomonas albilineans*, D2U8L0), CrSDH2 (*Chlamydomonas reinhardtii*, A8HX04), ScSDH2 (*Saccharomyces cerevisiae*, P21801).

The mutation site was located at the common region of OsSDH2-1 and SDH2-RPS14 (Supplementary Figure S3). However, due to the removal of the common region after being imported into mitochondria, functional RPS14 did not contain the mutation site. Hence, we carried out sequence similarity analysis with OsSDH2-1 and its homologs. The results showed that OsSDH2-1 had high identity with monocotyledons *Sorghum bicolor* (94.7%), *Zea mays* (93.6%) and *Triticum aestivum* (93.0%), medium identity with dicotyledons *Solanum lycopersicum* [SlSDH2-1 (84.3%) and SlSDH2-2 (71.5%)], *Arabidopsis thaliana* [AtSDH2-1 (71.4%) and AtSDH2-2 (73.6%)] and *Nicotiana tabacum* (72.1%) and lower identity with AtSDH2-3 (65.3%), OsSDH2-2 (55.5%) and microorganisms *Xanthomonas albilineans* (63.0%), *Chlamydomonas reinhardtii* (58.9%) and *Saccharomyces cerevisiae* (58.7%) (Supplementary Figure S4). Phylogenetic analysis showed that OsSDH2-1 and SDH2 homologs from other monocotyledons formed a clade, then formed a large clade with SDH2 homologs from dicotyledons except AtSDH2-3. By contrast, OsSDH2-2 and AtSDH2-3 formed a clade out of the large clade of other SDH2 homologs (Figure 6). These results suggested that OsSDH2-1 had more close relationship with SDH2 homologs from monocotyledons than those from dicotyledons and also had more close relationship with AtSDH2-1 and AtSDH2-2 than OsSDH2-2. 

### 2.4. The Mutant Phenotype of Lps1 Was Rescued by Wild Type LOC_Os08g02640 Gene

**Figure 7:**
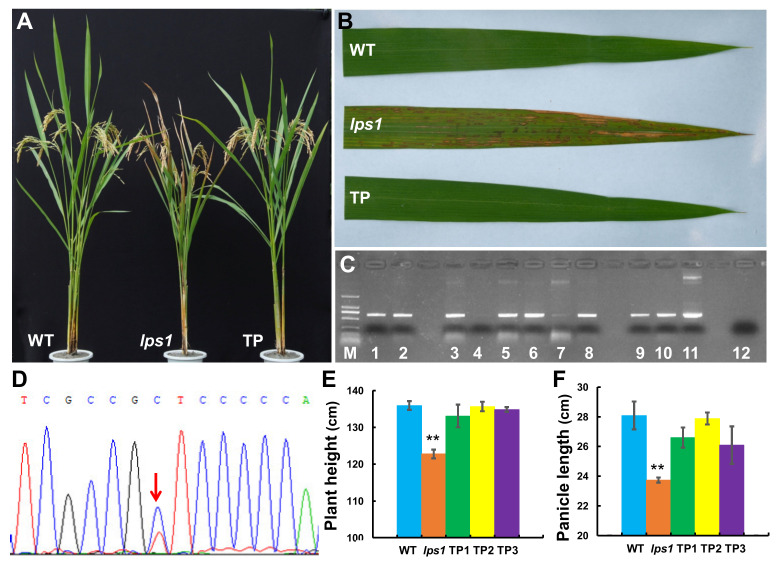
Complementation analysis of *lps1* mutant. (**A**) Phenotypes of wild type, *lps1* and positive transgenic plant (TP) at grain filling stage. (**B**) Flag leaf of wild type, *lps1* and TP plant at heading stage. (**C**) Identification of transgenic plants by PCR and electrophoresis. M: DL-2000 marker; 1-3, 5, 6 and 8–10: positive transgenic plants; 4 and 7: negative transgenic plants; 11: pCAMBIA1300-*OsSDH2-1* plasmid (PCR-positive control); 12: the *lps1* mutant (PCR-negative control). (**D**) Identification of transgenic plants by PCR and sequencing. The red arrow indicates the mutation site, showing double peaks in positive transgenic plants. (**E**) Plant height and (**F**) panicle length of wild type, *lps1* and TP plant at grain-filling stage. Error bars represent standard deviations of three independent biological replicates. The statistically significant differences were performed by Student’s *t* test. ** *p* < 0.01.

To further confirm the candidate gene for *lps1*, we constructed complementation vector and carry out genetic complementation experiment. First, the construct *pCAMBIA1300-LOC_Os08g02640* carrying a genomic fragment of 5993 bp encompassing 2523 bp of the 5’-upstream region, 1793 bp of the entire *LOC_Os08g02640* sequence and 1677 bp of the 3’-downstream region was generated. Then, the resulting construct was transformed into *lps1* mutant. Finally, 24 transgenic plants were obtained, and 19 positive transgenic plants were identified with PCR test and sequencing (Figure 7C,D). All positive transgenic lines showed normal phenotype as wild type, such as green leaves without premature senescence as well as normal plant height and panicle length (Figure 7A,B,E,F). These data suggested that *LOC_Os08g02640* gene rescued the mutant phenotype of *lps1* mutant, from which we concluded that the *LPS1* gene was indeed *LOC_Os08g02640*. 

### 2.5. Excessive Accumulation of Reactive Oxygen Species in Lps1 Leaves and Young Panicles

**Figure 8:**
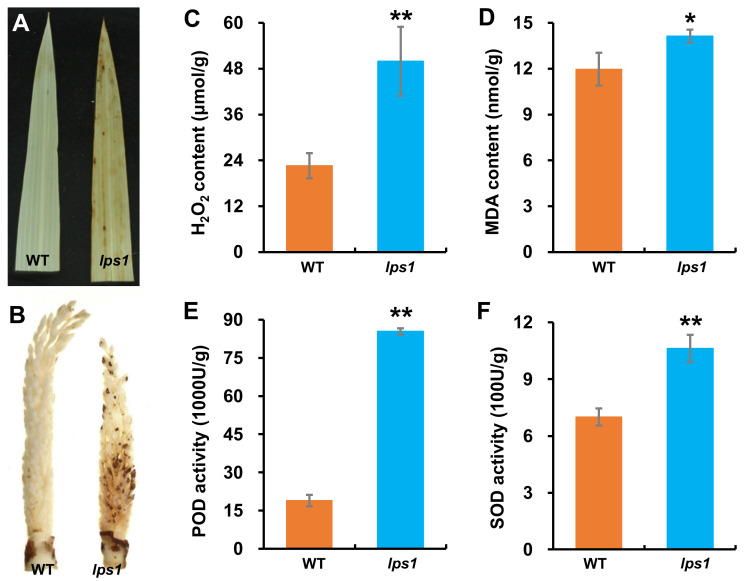
Detection and measurement of physiological and biochemical parameters associated with senescence. (**A**,**B**) 3,3′-diaminobenzidin (DAB) staining of leaves and young panicles, respectively. (**C**) H_2_O_2_ content. (**D**) Malondialdehyde (MDA) content. (**E**) Peroxidase (POD) activity. (**F**) Superoxide dismutase (SOD) activity. Error bars represent standard deviations of three independent biological replicates. The statistically significant differences were performed by Student’s *t* test. * *p* < 0.05 and ** *p* < 0.01.

It was reported that premature leaf senescence in rice mutants was induced by excessive accumulation of reactive oxygen species (ROS) [22,23,24]. Additionally, H_2_O_2_ is the main ROS in SDH-dependent ROS [8,25]. Thus, we detected levels of H_2_O_2_ in leaves of wild-type and *lps1* mutant plants. Firstly, 3,3′-diaminobenzidin (DAB) staining was carried out with leaves of *lps1* and wild type. As shown in Figure 8, *lps1* leaves were stained into light brown, while no dyeing was observed on leaves of wild type after DAB staining (Figure 8A). The DAB staining result indicated that *lps1* leaves accumulated hydrogen peroxide (H_2_O_2_). Due to smaller panicles and reduced seed-setting rate of *lps1* compared to those of wild type, we want to know whether ROS were also accumulated in young panicles. Then, DAB staining was performed with young panicles. The results showed that *lps1* young panicles were stained into light brown with dark brown spots on spikelets, while young panicles of wild type were still almost white after DAB staining (Figure 8B), indicating that excess H_2_O_2_ was accumulated in young panicles of *lps1*.

Furthermore, quantitative determinations were carried out with leaves of wild type and *lps1*. The results showed that contents of H_2_O_2_ and malondialdehyde (MDA) of *lps1* leaves were significantly more than those of wild type by 121.0 and 18.1%, respectively (Figure 8C,D), and the activities of superoxide dismutase (SOD) and peroxidase (POD) in *lps1* leaves were significantly higher than those of wild type by 51.6 and 351.0% (Figure 8E,F). MDA is the by-product of membrane lipid peroxidation induced by ROS [23]. ROS scavenging systems are induced to adjust ROS contents [24]. For example, SOD transforms O_2_.^-^ into H_2_O_2_ and O_2_, and POD converts H_2_O_2_ to H_2_O and O_2_ [26]. Therefore, more MDA content and higher increased SOD and POD activities indicated that more ROS was produced in *lps1* leaves compared with wild type.

### 2.6. Fertility of Pollen Grains Was Reduced in Lps1

**Figure 9:**
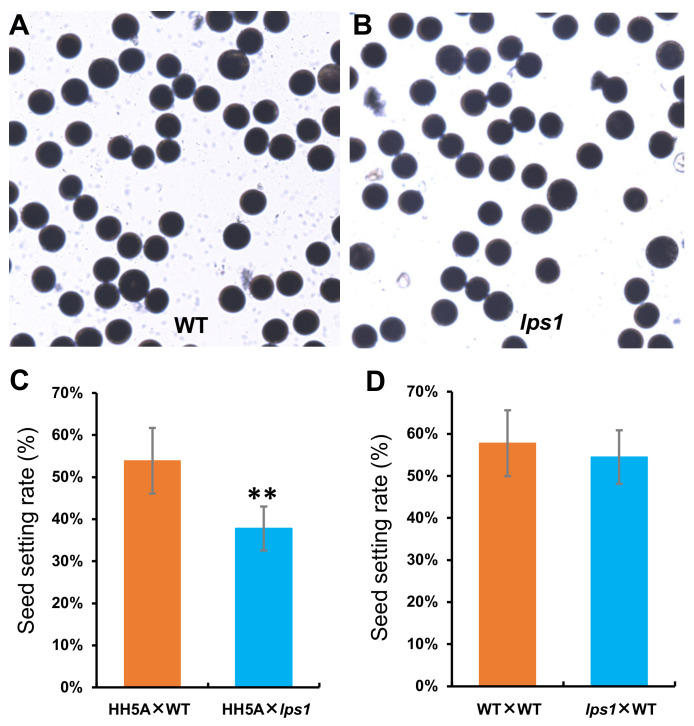
Detection of male and female fertility of *lps1* mutant. (**A**,**B**) Pollen grains of the wild type and *lps1* were stained with I_2_-KI solution, respectively. (**C**) The wild type and *lps1* were used as pollen donors and crossed with cytoplasmic male sterile line Huihe 5A (HH5A), respectively. (**D**) The wild type and lps1 were artificially emasculated and then were pollinated by using the other normal wild-type plants, respectively. Error bars represent standard deviations of three independent biological replicates. The statistically significant differences were performed by Student’s *t* test. ** *p* < 0.01.

To find the underlying reason for low seed-setting rate of *lps1*, we first stained its pollen grains with I_2_-KI solution. As shown in Figure 9A,B, there was no obvious difference in dyeing of pollen grains between *lps1* and its wild type. At the same time, *lps1* and the wild type were used as pollen donors and crossed with cytoplasmic male sterile line Huihe 5A, respectively. The result showed that the seed-setting rate of (Huihe 5A × *lps1*) combination was significantly reduced by 16.1%, compared with that of (Huihe 5A × WT) combination (Figure 9C), suggesting that male fertility of *lps1* was affected by the mutation in *SDH2-1* gene. On the other hand, *lps1* and its wild type were artificially emasculated and then were pollinated by using the other normal wild-type plants, respectively. The result showed that there was no significant difference in the seed-setting rates between (*lps1* × WT) and (WT × WT) combinations (Figure 9D), suggesting that female fertility of *lps1* was not affected by the mutation in *SDH2-1* gene. Therefore, the reduction in the seed-setting rate could be resulted from reduced fertility of pollen grains in *lps1* mutant.

### 2.7. OsSDH2-1 Was Located in Mitochondria

**Figure 10:**
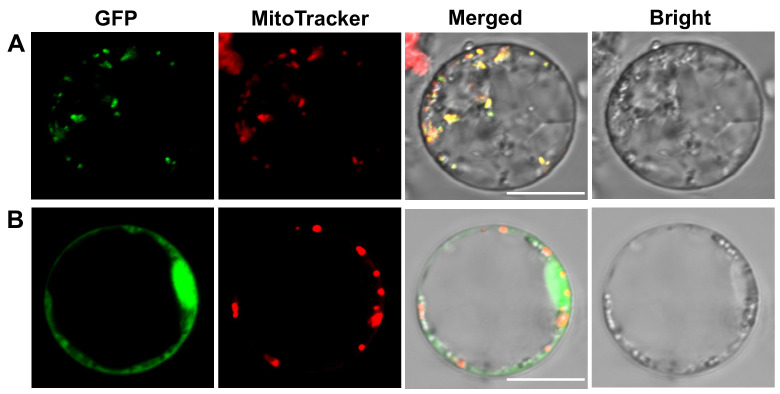
Subcellular localization of OsSDH2-1 protein. (**A**) OsSDH2-1-GFP (Green fluorescent protein) fusion protein. (**B**) Empty GFP. Green fluorescence indicates the signals from GFP protein, and red fluorescence shows the signals from mitochondria stained with MitoTracker Red CMXRos. Bars = 5 μm.

A mitochondrial transit peptide was predicted in OsSDH2-1 by TargetP-2.0 server [27]. To confirm the actual subcellular localization of OsSDH2-1, the full-length coding sequence of *OsSDH2-1* was inserted into pCAMBIA2300-35S-*GFP* vector. Then, the pCAMBIA2300-35S-*OsSDH2-1*-*GFP* was transformed into rice protoplasts prepared from etiolated seedlings, and MitoTracker Red CMXRos was used as a marker for mitochondria. As control group, pCAMBIA2300-35S-*GFP* was also co-transformed into rice protoplasts. The results of confocal microscope showed that green fluorescence from OsSDH2-1-GFP fusion protein was overlapped with red fluorescence from MitoTracker Red CMXRos (Figure 10A). Conversely, green fluorescence from GFP protein did not overlap with red fluorescence from MitoTracker Red CMXRos in the control group (Figure 10B). Our results identified that OsSDH2-1 was located in mitochondria, being consistent with its functions.

### 2.8. LOC_Os08g02640 Gene Was Ubiquitously Expressed in Rice

**Figure 11:**
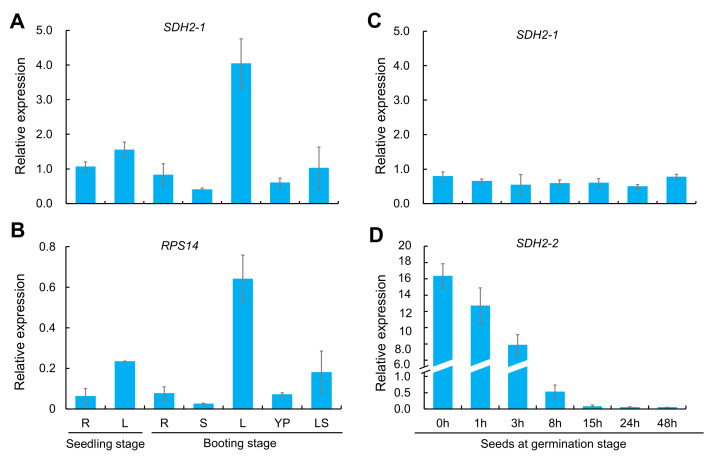
Spatio-temporal expression pattern of *OsSDH2-1*, *RPS14* and *OsSDH2-2* in the wild type. (**A**,**B**) Expression pattern comparison between *OsSDH2-1* and *RPS14* in young and adult plants. R: root, S: stem, L: leaf blade, YP: young panicle, and LS: leaf sheath. (**C**,**D**) Expression pattern comparison between *OsSDH2-1* and *OsSDH2-2* in seeds at germination stage, respectively. *Actin 1* was examined as the internal control. Error bars represent standard deviations of three independent biological replicates.

*LOC_Os08g02640* has two alternative splice forms: *OsSDH2-1* and *RPS14*. To compare expression difference of *OsSDH2-1* and *RPS14*, we firstly detected amplification efficiency of *OsSDH2-1* and *RPS14* qRT-PCR specific primers with the wild-type genomic DNA. The result showed the same amplification efficiency of *RPS14* and *OsSDH2-1* RT-PCR (Supplementary Figure S5). Then, qRT-PCR analysis was conducted to study expression patterns of *OsSDH2-1* and *RPS14* with RNAs extracted from various organs of the wild type at seedling and booting stages. As shown in Figure 11, *OsSDH2-1* and *RPS14* were expressed in all detected organs and exhibited the similar expression patterns with the highest expression in top-one leaves at booting stage and the low expression in roots, stems and young panicles (Figure 11A,B). Considering the amplification efficiency of genomic DNAs, mRNA level of *OsSDH2-1* was much higher than that of *RPS14*, implying that mRNAs of *OsSDH2-1* and *RPS14* might be alternatively spliced from the mRNA precursor of *SDH2-RPS14* in a relatively fixed ratio.

**Figure 12:**
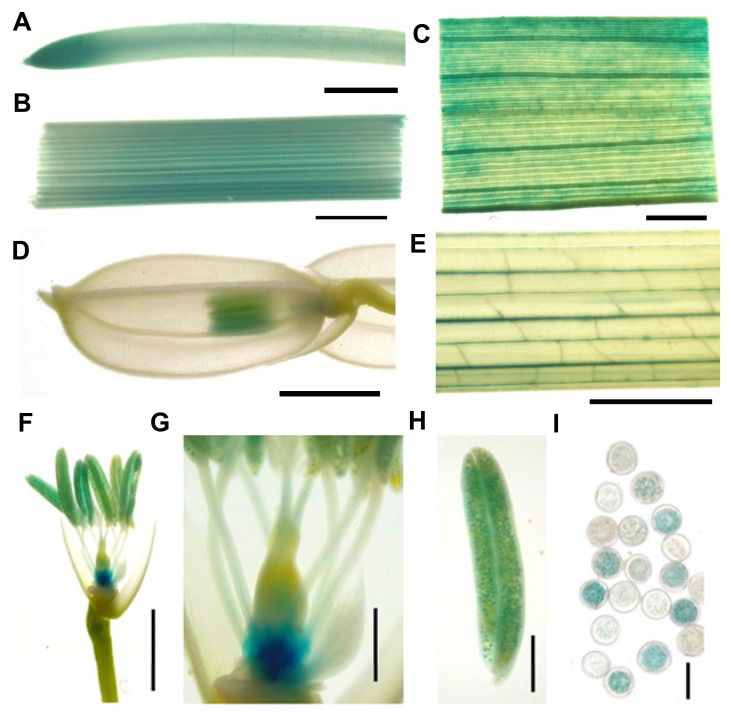
Expression pattern revealed by GUS staining; 2523 bp promoter sequence of *LPS1* was ligated into the binary vector pCAMBIA1391Z upstream of the GUS reporter gene. (**A**) Root. (**B**) Stem. (**C**) Leaf blade. (**D**) Young panicle. (**E**) Leaf sheath. (**F**) Pistils and stamens. (**G**) Ovary, filament and style. (**H**) Anther. (**I**) Pollens. (**A–F**): Bar = 2 mm; (**G–H**): Bar = 0.5 mm.; (**I**): Bar = 50 μm.

To further detect the promoter activity of *LOC_Os08g02640* gene, β-glucuronidase (GUS) staining was carried out with tissues and organs from transgenic plants transformed with pCAMBIA1391Z vector including 2523 bp promoter sequence of *LOC_Os08g02640* gene and *GUS* reporter gene. As shown in Figure 12, *LOC_Os08g02640* gene was ubiquitously expressed in all tested tissues and organs (Figure 12A–E). Deep GUS staining in root tips and anthers indicated that *LOC_Os08g02640* gene was highly expressed in those tissues (Figure 12A,D). In addition, a sharply reduced seed-setting rate of *lps1* and deep GUS staining in anthers promoted us to observe the GUS signals in spikelet in more detail. Interestingly, we observed very strong GUS signal at the base of ovary (Figure 12F) and strong GUS staining in a part of pollens (Figure 12H,I). Besides, low GUS activity was detected in vascular tissues of filaments and styles (Figure 12G). Perhaps, a high expression level of *LOC_Os08g02640* gene in floral organs could be related to the low seed-setting rate of *lps1*.

In addition, we also examined expression pattern of *OsSDH2-1* and *OsSDH2-2* in seeds at an early germination stage. As shown in Figure 11, *OsSDH2-2* was expressed in seeds at an early germination stage (before 8 h) with the highest expression level in dry seeds (Figure 11D) and nearly not expressed in other tissues and organs (Supplementary Figure S6). While *OsSDH2-1* was expressed in the whole stage of seed germination (Figure 11C).

### 2.9. Senescence- and ROS Detoxification-Associated Genes Were Upregulated in Lps1 Leaves

**Figure 13:**
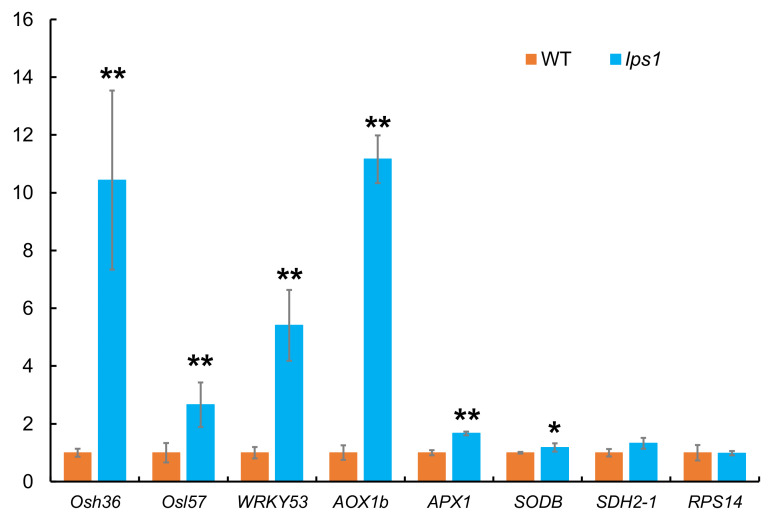
Expression analysis of senescence-associated and ROS detoxification-associated genes in flag leaves of wild type and lps1 mutant at heading stage by qRT-PCR. The expression level of each gene in the wild type was set to 1.0, and those in *lps1* mutant were calculated accordingly. Error bars represent standard deviations of three independent biological replicates. The statistically significant differences were performed by Student’s *t* test. * *p* < 0.05 and ** *p* < 0.01.

*lps1* mutant showed premature senescence leaf and accumulated reactive oxygen species, so we examined expression difference of three senescence-associated genes (SAGs), three ROS detoxification-associated genes (RDAGs) and *OsSDH2-1* and *RPS14* between *lps1* and wild type. Among these genes, *Osh36* and *Osl57* genes are SAGs involving in amino acid and fatty acid metabolism in senescence [28]. *OsWRKY53* is a transcription factor regulating the expression of many SAGs and is induced by ROS [29]. *AOX* (*Aox1b*), *APX* (*APX1*) and *SOD* (*SDOB*) are ROS-scavenging genes [30]. As shown in Figure 13, expression levels of SAGs and RDAGs were all significantly upregulated in the senescing leaves of *lps1* compared to those of wild type, and especially expression levels of *Osh36*, *OsWRKY53* and *Aox1b* sharply upregulated by about 10-, 6- and 11-fold, respectively. Besides, there was no significant difference on the transcript levels of *OsSDH2-1* and *RPS14* between *lps1* and wild type (Figure 13). 

## 3. Discussion 

*SDH2* have been widely studied in microorganisms *Escherichia coli* [31], plants Arabidopsis [12,13,14,16,32,33] and tomato [17], animal nematode [34] and humans [35]. However, it is yet unknown whether *SDH2* genes are involved in leaf senescence and yield formation in higher plants. In this study, we isolated a mutant (*lps1*, *indica* background) showing late premature senescence, small panicles, and low seed-setting rate (Figure 1C,D and Figure 2F). The mutant displayed decreased photosynthetic capacity of leaves, reduced fertility of pollen grains, and excessively accumulated ROS in leaves and young panicles (Figure 5, Figure 8 and Figure 9). Gene mapping and MutMap analysis suggested that a single-base substitution occurred in candidate gene *LOC_Os08g02640*, which caused an amino acid substitution in the encoded OsSDH2-1 protein (Supplementary Figure S2). Furthermore, complementation analysis confirmed that the mutation of the *LOC_Os08g02640* gene was responsible for mutant phenotype in *lps1* (Figure 7). Therefore, our data revealed an important role of *SDH2-1* in leaf senescence and yield formation in rice.

Intriguingly, *LOC_Os08g02640* gene encodes two proteins, OsSDH2-1 and chimeric SDH2-RPS14, by alternative splicing [19] (Supplementary Figure S3). The additional polypeptide in chimeric SDH2-RPS14 is identical to the corresponding sequence of OsSDH2-1 and provides the mitochondrial signal peptide for RPS14. When SDH2-RPS14 is imported into mitochondria, the additional polypeptide is removed and degraded, and RPS14 is assembled into mitochondria ribosomes in rice and maize [19,21]. In our study, the mutation site was located at the common region of OsSDH2-1 and SDH2-RPS14, which will be degraded in chimeric SDH2-RPS14 after being imported into mitochondria (Supplementary Figure S3). On the other hand, there was no significant difference in *RPS14* expression level between wild type and *lps1* (Figure 13). According to these data, we considered that the mutant phenotype of *lps1* should be caused by the mutation in OsSDH2-1, and the mutation in *lps1* could not affect biological function of RPS14 protein.

T-DNA insertion in *SDH2-1* or *SDH2-2* does not cause abnormal phenotype in *Arabidopsis thaliana*, due to the functional redundancy relationship between *SDH2-1* and *SDH2-2* [14,15]. However, double *sdh2-1*/*sdh2-2* homozygous mutants could not be obtained, implying the double homozygous mutants might be lethal [15]. *SDH2-3* is specifically expressed during seed development, and its knockout leads to a delay of seed germination [13,16]. Nevertheless, tomato *SDH2-2* knock-down lines show increased photosynthetic rate and enhanced growth due to markedly elevated transpiration rate and stomatal conductance [17]. In our study, *lps1* mutant exhibited the phenotypes different from Arabidopsis and tomato *sdh2* mutants, such as late premature senescence (Figure 1B,C), decreased net photosynthetic rate and stomatal conductance (Figure 5), small panicle size (Figure 1D and Figure 2E) and reduced seed-setting rate (Figure 2F). The new phenotypes of *lps1* might derive from the different suffering of SDH2 genes, such as a single-base substitution in rice and antisense inhibition in tomato. Another possible explanation is that the functions of SDH2 proteins could have diversified in different plant species and even in the same species. 

Semiquantitative RT-PCR analysis showed that *AtSDH2-1* and *AtSDH2-2* had similar expression patterns in roots, stems, leaves, inflorescences and flowers, with the lowest and highest mRNA expressions in leaves and flowers, respectively [14]. RNA in situ hybridization and histochemical GUS staining showed that the expressions of *AtSDH2-1* and *AtSDH2-2* were highly expressed in floral and inflorescence meristems, in sex organ primordia, and in anthers [14]. However, they were differentially expressed in root tips; for example, *AtSDH2-1* transcripts were barely detected, but *AtSDH2-2* was strongly expressed in root tips [14]. In this study, qRT-PCR analysis revealed that *OsSDH2-1* was ubiquitously expressed in roots, stems, leaves, young panicles and leaf sheaths, with the most abundance mRNA in top-one leaves at the booting stage (Figure 11A). Histochemical GUS staining revealed that *OsSDH2-1* was highly expressed in root tips, ovary and anthers (Figure 12). These results suggested that *OsSDH2-1* simultaneously possessed expression patterns of *AtSDH2-1* and *AtSDH2-2*, implying that *OsSDH2-1* could have the functions of the two Arabidopsis genes. Nevertheless, different to *AtSDH2-1* and *AtSDH2-2*, *OsSDH2-1* was expressed with higher transcript levels in leaves, which may be one of the reasons of premature leaf senescence phenotype of *lps1*.

SDH2 harbors three iron-sulfur clusters: 2Fe-2S, 4Fe-4S and 3Fe-4S [5], which are responsible for the electron transport from FAD in SDH1, and are indispensable for SDH, participating the electron transport chain. Three cysteine-rich clusters in the SDH2 amino acid sequence provide cysteine residues to fix the three iron-sulfur clusters [19,36] (Supplementary Figure S4). Previous studies have reported that thenoyltrifluoroacetone (TTFA), as a specific and noncompetitive inhibitor for SDH, could induce ROS release and accumulation [8]. Additionally, TTFA is found to bind in the proximity of 3Fe-4S of SDH2 to inhibit electron transport [4]. In our study, the mutation site of OsSDH2 in *lps1* mutant was located near the 2Fe-2S cluster and was highly conserved between prokaryote and eukaryote (Supplementary Figure S4), suggesting that this site was important to SDH2 function. Based on the above data, the mutation site in *lps1* mutant probably interrupted the electron transport from SDH1 to SDH3/4 and caused ROS release and accumulation. Therefore, excess ROS accumulation could cause upregulation of senescence-associated genes (SAGs) and ROS detoxification-associated genes (RDAGs) and finally led to late premature senescence phenotype of *lps1* mutant. 

Higher accumulation of ROS over a certain level often leads to male sterility phenotypes, such as defective anther development, aborted pollen grains and failure of fertilization in rice, Arabidopsis and tomato [37,38,39]. In rice *degenerated panicle and partial sterility 1* (*dps1*) mutant, panicle apical degeneration and abnormal anther were caused by ROS-mediated enhanced cell death [40]. In this study, the *lps1* mutant also displayed obvious reduction in grain number per panicle and seed-setting rate relative to its wild type (Figure 2E,F). Detection of male and female fertility suggested that the reduction in seed-setting rate could be resulted from the reduced fertility of pollen grains in the *lps1* mutant. On the other hand, DAB staining showed that young panicles of *lps1* accumulated excess H_2_O_2_. Taken together, excessive accumulation of H_2_O_2_ could affect panicle development and pollen function and finally resulted in small panicles, low seed-setting rate and severely decreased yield in the mutant.

## 4. Materials and Methods

### 4.1. Plant Materials and Growth Conditions

The late premature senescence mutant *lps1* was isolated from a mutant pool of main restorer line 188R (*indica*) via EMS mutagenesis. *lps1* mutant was crossed with the wild-type 188R to perform genetic analysis and was crossed with Nipponbare (*japonica*) to map the *lps1* locus. All plant materials were grown in natural growing season in paddy fields at Chengdu, Sichuan, China.

### 4.2. Measurement of Photosynthetic Pigments and Capacity

Photosynthetic pigments were extracted from the top-one leaves at booting stage and the flag leaves at heading stage with 80% acetone in the dark for 48 h at 4 °C and then the contents of pigments were determined at 663, 646 and 470 nm using a UV-visible spectrophotometer (BioMate 3S, Thermo Scientific, Waltham, MA, USA) according to Lichtenthaler and Wellburn [41]. Net photosynthetic rate and stomatal conductance of leaves at the same stages were investigated by a por figure photosynthetic apparatus (Li-6400, Li-COR Inc., Lincoln, NE, USA). Each leaf from wild type and *lps1* mutant at the same stage was measured right after each other in a short period. All measurements were conducted under the environmental control settings: a photon flux density of 1200 μmol m^−2^ s^−1^, temperature of 30 °C and CO_2_ concentration of 400 ppm.

### 4.3. Transmission Electron Microscope Analysis

Chloroplast ultrastructure was observed by transmission electron microscope. The middle sections of flag leaves showing premature senescence phenotype at leaf tip of *lps1*, and the corresponding leaf sections of wild type were harvested and fixed in the fixation solution (3% glutaraldehyde), and then refixed with 1% osmium tetroxide. After being treated with a series ethanol (30, 50, 70, 80, 90, 95 and 100%), Epon 812, uranyl acetate and Reynolds’ lead citrate, the samples were observed with a transmission electron microscope (H-600, Hitachi, Tokyo, Japan).

### 4.4. Gene Mapping and Candidate Gene Analysis of LPS1

For gene mapping, 116 plants showing late premature senescence phenotype were collected from F_2_ mapping population crossed between *lps1* mutant and Nipponbare (*japonica*). Over 300 microsatellite markers dispersing in the rice genome were selected to perform the preliminary mapping. Then, new InDels markers were developed based on the genomic difference between *japonica* Nipponbare and *indica* R498 to further narrow the region. All the primers used in the mapping were listed in Supplementary Table S1. 

To identity candidate gene of *LPS1*, rice leaves from 30 plants displaying late premature senescence in the (*lps1* × 188R) F_2_ population were cut into small pieces and mixed in an equal ratio. Subsequently, high-throughput sequencing and MutMap analysis [42] were performed to detect the single nucleotide polymorphisms (SNPs) between genomes of *lps1* mutant and 188R with R498 genome as the reference genomic sequence.

### 4.5. Complementation Analysis

To construct complementation vector, the full-length genomic sequence of *LPS1* with its promoter were amplified from wild type using specific primers (F1: GAGCTCGGTACCCGGGGATCCGTAGGAGCTACAGCATGT; R1: CAGGTCGACTCTAGAGGATCCTTACGGAGAATGCTTGTT). The amplification products containing the overlapping extensions at 5′-terminus were inserted into the *Bam*HI linearized pCAMBIA1300 vector with the ClonExpress™ II One Step Cloning Kit (Vazyme, Nanjing, China). This resulting pCAMBIA1300-*LPS1* construct was transformed into *lps1* mutant by Agrobacterium-mediated method. Identification of positive transgenic lines were conducted by amplifying and electrophoresis with specific primers (F2: GTGATTGCTCTGTCCTAT on the 3′UTR of *LPS1*, R2: CGACGGCCAGTGCCAAGC on pCAMBIA1300 vector), and by amplifying and sequencing with specific primers (F3: CGTCGACGAAGCCCCACCTC, R3: GAAGGTGGTGGTATACCTGT).

### 4.6. Sequence Analysis 

Homolog sequences of LPS1 were obtained by BlastP in National Center for Biotechnology Information (NCBI) and Universal Protein (UniProt, www.uniprot.org/, access on 3rd April 2020). Multiple sequence alignment was carried out using DNAMAN Software. The relationship among homolog sequences was reconstructed with MEGA using neighbor-joining method after alignment with ClustalW.

### 4.7. Subcellular Localization 

TargetP-2.0 server was used to predict signal peptide and subcellular localization [27]. To determine the subcellular localization of LPS1 protein, the full-length coding sequences were isolated from wild type with specific primers (F4: GGACAGGGTACCCGGGGATCCATGGCCGCCGCCGCCCTCCT, R4: AGTGTCGACTCTAGAGGATCCTGAGGCCTCGAGCTGGAGCT). The amplification products containing the overlapping extensions at 5′-terminus were inserted into the *Bam*HI linearized pCAMBIA2300 vector with the ClonExpress™ II One Step Cloning Kit (Vazyme, Nanjing, China). The construct pCAMBIA2300-35S-*LPS1*-GFP was transformed into rice protoplasts prepared from etiolated seedlings according to the method of Zhang et al. [43]. Before imaging, the protoplasts were incubated with 200 nM MitoTracker Red CMXRos (Invitrogen, Carlsbad, CA, US), a mitochondria dye, for 15 min at 37 °C. Then, a laser-scanning confocal microscope (Nikon A1, Nikon, Japan) was used to detect the fluorescence signals in rice protoplasts.

### 4.8. Detection of Reactive Oxygen Species

Flag leaves at heading stage and young panicles at booting stage were collected to detect the accumulation of hydrogen peroxide (H_2_O_2_) with 3,3′-diaminobenzidin (DAB). Furthermore, contents of hydrogen peroxide (H_2_O_2_) and malondialdehyde (MDA), and activities of antioxidant enzymes including peroxidase (POD) and superoxide dismutase (SOD) in flag leaves at heading stage were measured. All detection and measurement were conducted with commercial kits from Solarbio (Beijing, China) following the manufacturer’s instructions. 

### 4.9. Detection of Male and Female Fertility

Pollen grains of *lps1* and its wild type were stained with 1% I_2_-KI solution and then were observed by using a microscope, respectively. To further detect male and female fertility of *lps1*, two crossing experiments were conducted: (1) *lps1* and the wild type were used as pollen donors and crossed with cytoplasmic male sterile line “Huihe 5A”, respectively; (2) *lps1* and the wild type were artificially emasculated and then were pollinated by using the other normal wild-type plants (188R), respectively. At approximately 15 days after pollination, the outcrossing seed-setting rates were investigated.

### 4.10. Histochemical Staining Analysis

To investigate the tissue expression, 2523 bp promoter sequence (upstream from start codon ATG) of *LPS1* was amplified with specific primers (F5: TGGCTGCAGGTCGACGGATCCGTAGGAGCTACAGCATGT; R5: CCAGTGAATTCCCGGGGATCCCGCGGCGGATTTGGGGTT) and then fused into pCAMBIA1391Z digested with *Bam*HI. The resulting *LPS1_Pro_*::*GUS* construct was transformed into *japonica* variety ZH11 by Agrobacterium-mediated method. The positive *LPS1_Pro_*::*GUS* reporter lines were identified by PCR with specific primers (F6: AACTCTCTAGGCTATTCC on the 3′-end of *LPS1* promoter, R6: GTGGACTCCTCCCTAGGCTTC on pCAMBIA1391Z vector). Organs and tissues including root, stem, leaf blade, leaf sheath and young panicle were collected and soaked in the GUS staining solution (Coolaber, Beijing, China) at 37 °C for 24 h. After destained with 70% alcohol, the samples were photographed by a stereomicroscope (S9i, Leica, Heerbrugg, Switzerland).

### 4.11. Quantitative RT-PCR Analysis

Total RNA was extracted from various rice organs using Total RNA Extraction Reagent (R401-01, Vazyme, Nanjing, China). The first-strand cDNA was obtained using HiScript II Q RT SuperMix (R223-01, Vazyme). Quantitative RT-PCR was carried out with 2 × SYBR Green qPCR Master Mix (B21202, Bimake, Houston, TX, USA) in the real-time PCR system (qTOWER^3^ G, analytikjena, Jena, Germany). Expression patterns of *OsSDH2-1*, *RPS14* and *OsSDH2-2* were detected with cDNA of roots and leaves at seedling stage, and roots, stems, leaves, young panicles and leaf sheaths at booting stage or seeds after 0, 1, 3, 8, 15, 24 and 48 h imbibition. Expression differences of three senescence-associated genes (*Osh36*, *Osl57* and *OsWRKY53*) and three ROS detoxification-associated genes (*AOX1b*, *APX1* and *SODB*) were quantified between wild type and *lps1* mutant. *Actin1* was used as an internal control, and all primers were listed in Supplementary Table S1.

### 4.12. Statistical Analysis

Excel in Office 2016 was used to perform the statistical analysis. All experiments were repeated independently for three replicates, and data were subjected to statistical analysis using the Student’s *t*-test with a *p*-value less than 0.05 or 0.01 considered significant.

## Data Availability

The data presented in this study are available in the article or supplementary material.

## Supplementary Materials

Supplementary materials can be found at https://www.mdpi.com/1422-0067/22/1/157/s1. Figure S1, Comparison of grain length and width between WT and *lps1*. Figure S2, Preliminary mapping of the *lps1* locus. Figure S3, Schematic representation of *LOC_Os08g02640* gene, *RPS14* cDNA and *OsSDH2-1* cDNA. Figure S4, Homolog sequences of OsSDH2-1. Figure S5 Detection of PCR amplification efficiency of OsSDH2-1 and RPS14 RT-PCR specific primer in wild-type genomic DNA. Figure S6, Detection of *SDH2-2* expression in various organs of wild type. Table S1, Primers used in the study.

## Abbreviations

ROS	Reactive oxygen species
SDH	Succinate dehydrogenase
TCA	Tricarboxylic acid
ETC	Electron transport chain
EMS	Ethyl methanesulfonate
Chl	Chlorophyll
