# Supplementary material for: LPS1, Encoding Iron-Sulfur Subunit SDH2-1 of Succinate Dehydrogenase, Affects Leaf Senescence and Grain Yield in Rice
Source: Int J Mol Sci. 2020 Dec 25;22(1):157. doi: 10.3390/ijms22010157 (PMC7795075; doi:10.3390/ijms22010157)
Supplement: Supplementary file 1 [file ijms-22-00157-s001.pdf]

Supplementary Material

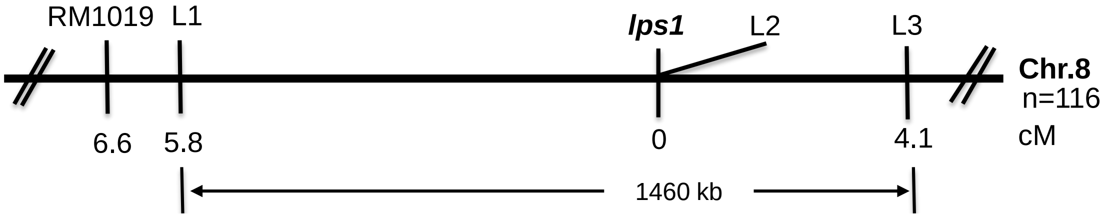

Figure S1 Preliminary mapping of the *lps1* locus.

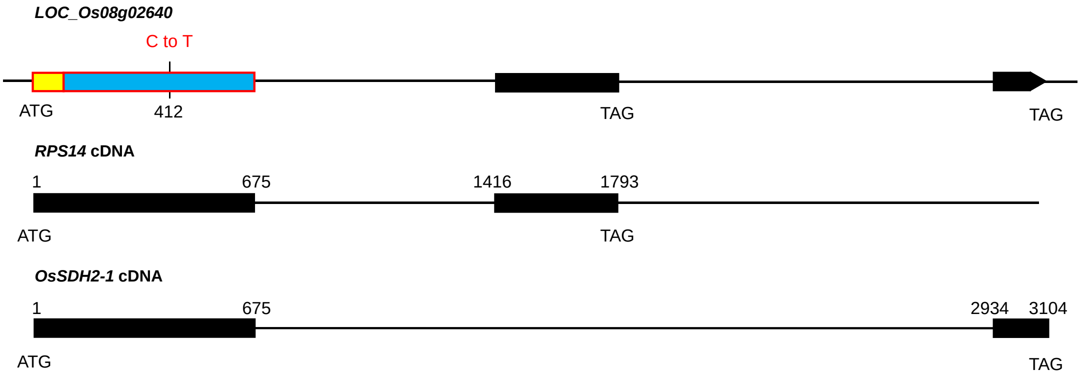

Figure S2 Schematic representation of *LOC_Os08g02640* gene, *RPS14* cDNA and *OsSDH2-1* cDNA. The substitution (C to T) happened at position 412 in genome sequence of *LOC_Os08g02640* gene. Red box shows the common region of *OsSDH2-1* and *RPS14* cDNAs, in which yellow block indicates the sequence encoding mitochondrial signal peptide and blue block indicates the sequence encoding a peptide specifically belonged to functional SDH2.

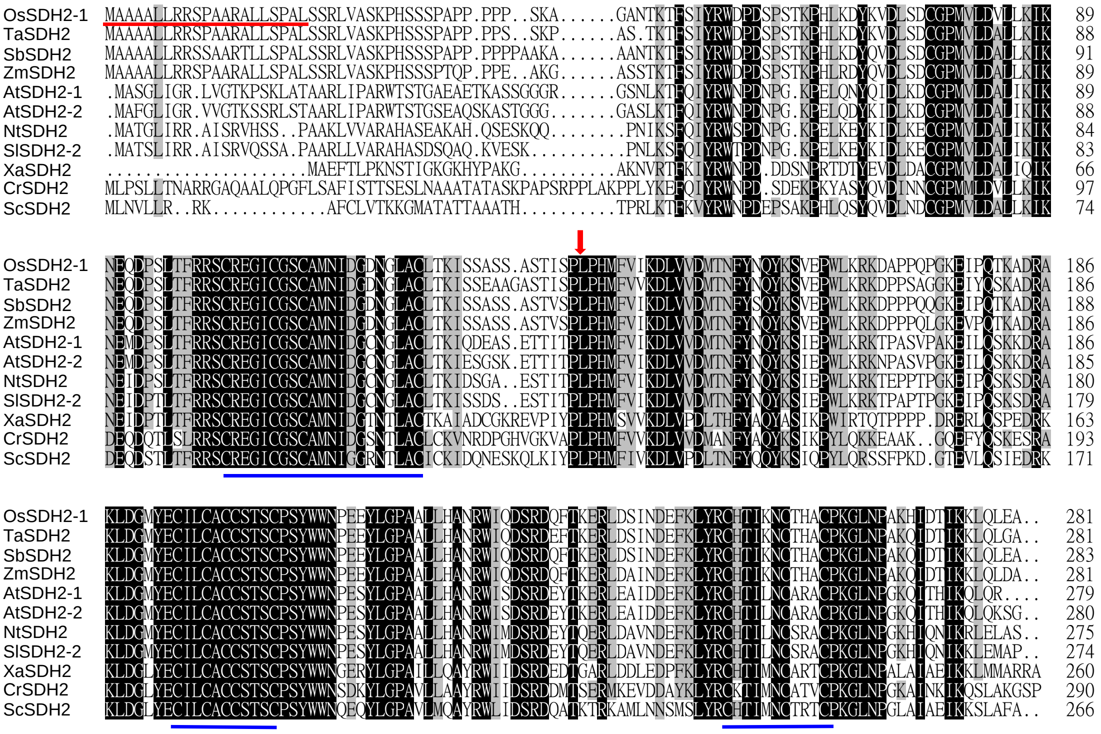

Figure S3 Homolog sequences of OsSDH2-1. Black shades indicate the identical residues and gray shades indicate the similar residues (≥75% identical). The red arrow indicates the mutation site of OsSDH2-1 in the *lps1* mutant, the blue lines indicate the cysteine clusters which were ligands to the iron-sulfur clusters, the red line indicates the mitochondrial signal peptide predicted from TargetP 1.1 Server. Accession numbers for the respective protein sequences are as Fig. 6.

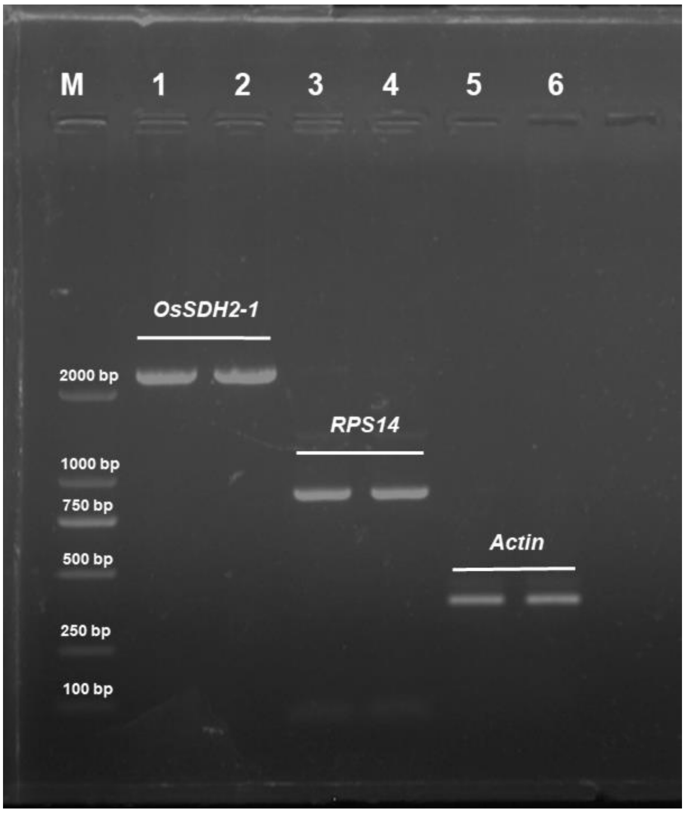

Figure S4 Detection of PCR amplification efficiency of *OsSDH2-1* and *RPS14* qRT-PCR specific primer in wild-type genomic DNA.

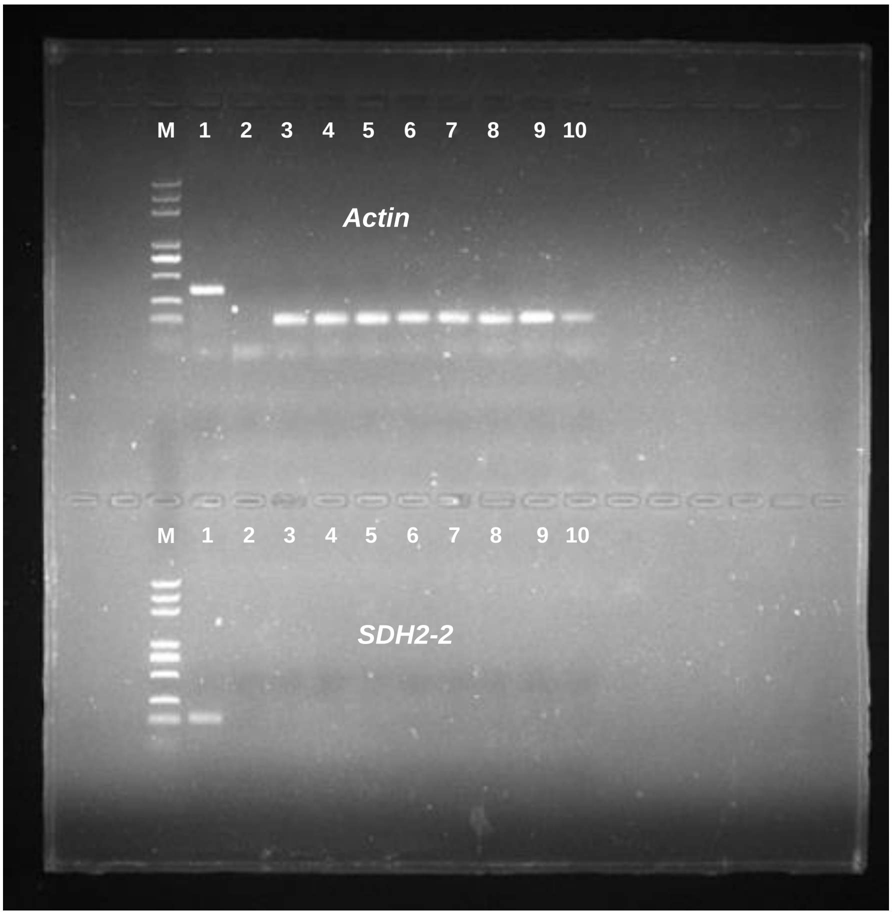

Figure S5 Detection of *SDH2-2* expression in various organs of wild type. M: DL-5000 marker; 1: positive control (genomic DNA); 2: negative control; 3: seeds after 48-hours imbibition; 4-5: root and leaf at seedling stage; 6-10: root, stem, leaf, young panicle and leaf sheath at booting stage.

Table S1 Primers used in the study.

Primers	Forward primer	Reverse primer	References
RM1019	GTTTGAACAGTAGGACTTGT	AGAACATCTCACACTTCTCT	[43]
L1	TGAAATCGGGATATGAG	TACTAGGAGCACGTTGC	
L2	CATTCCAACACTAGGCTCA	TCAATTCTTCGGTTCCAC	
L3	CGCCCATTAGTTCTCCGTAT	GCAGCGAAAGCACTGGAA	
OsSDH2-1	CATGTACGAGTGCATCCT	TCCTTAGTGAACTGGTCA	
OsSDH2-2	TCGTGCTCCATGTGCATC	CCTCACGACGTACATGTG	
RPS14	TCTGCGCCTGCTGCTCCA	GATGTGCATGAACATGTT	
Osh36	AACGCATTTGTGGTTGGCTC	TCAACTTTGGCCGGTGTCTT	[30]
Osl57	ACCCTAAAGTAAATGAAGTC	CCTGCTCTTGTCTTGTTA	[30]
OsWRKY53	GAGCGACATCGACATCCT	TTGTGCTTGCCCTCGTAG	[44]
AOX1b	CCTGCTCAGTTCATCACCATCA	GCATAAAACGGAGTGACAATAGC	[30]
APX1	AGGTGCCACAAGGAAAGATCTGGT	TCAGCAGGGCTTTGTCACTAGGAA	[30]
SODB	TCCGCCGTATAAACTTGATGCCCT	TGGGTTGCCGTTGTTGTATGCTTC	[30]
